# NETosis in Cancer – Platelet–Neutrophil Crosstalk Promotes Tumor-Associated Pathology

**DOI:** 10.3389/fimmu.2016.00373

**Published:** 2016-09-21

**Authors:** Anna-Karin Olsson, Jessica Cedervall

**Affiliations:** ^1^Science for Life Laboratory, Department of Medical Biochemistry and Microbiology, Biomedical Center, Uppsala University, Uppsala, Sweden

**Keywords:** cancer, neutrophil extracellular traps, neutrophils, platelets

## Abstract

It has become increasingly clear that circulating immune cells in the body have a major impact on cancer development, progression, and outcome. The role of both platelets and neutrophils as independent regulators of various processes in cancer has been known for long, but it has quite recently emerged that the platelet–neutrophil interplay is yet a critical component to take into account during malignant disease. It was reported a few years ago that neutrophils in mice with cancer have increased propensity to form neutrophil extracellular traps (NETs) – web-like structures formed by externalized chromatin and secreted proteases. The initial finding describing this as a cell death-associated process has been followed by reports of additional mechanisms for NET formation (NETosis), and it has been shown that similar structures can be formed also without lysis and neutrophil cell death as a consequence. Furthermore, presence of NETs in humans with cancer has been verified in a few recent studies, indicating that tumor-induced NETosis is clinically relevant. Several reports have also described that NETs contribute to cancer-associated pathology, by promoting processes responsible for cancer-related death such as thrombosis, systemic inflammation, and relapse of the disease. This review summarizes current knowledge about NETosis in cancer, including the role of platelets as regulators of tumor-induced NETosis. It has been shown that platelets can serve as inducers of NETosis, and the platelet–neutrophil interface can therefore be an important issue to consider when designing therapies targeting cancer-associated pathology in the future.

## Tumor-Induced Manipulation of the Immune System

Cancer development and progression is driven by complex interactions between neoplastic cells and non-malignant host cells. The tumor-promoting effects of the host cells often represent normal, or even essential, physiological functions that have been “hi-jacked” by the tumor microenvironment. A prominent example is platelet activation, which is required for wound healing and to prevent bleeding due to injury, while the same mechanism contributes to disease progression and mortality in individuals with cancer (1). Similarly, cells of the innate immune system that normally serve as an essential defense against infections can be modulated during malignancy to become promoters of disease. This phenomenon has been extensively studied especially for macrophages, where tumor progression is paralleled with a phenotypic switch from a tumor-suppressing classical M1-like subtype to a tumor-promoting M2-like macrophage. These M2 macrophages often represent the majority of immune cells in a solid tumor and contribute to tumor progression by immunosuppressive and pro-angiogenic mechanisms (2). Novel data indicate that neutrophil function is altered in a similar way during malignant disease. Neutrophils have an indispensible role as a first-line defense to combat infectious disease, a function mediated by phagocytosis and secretion of antimicrobial peptides (3). However, in individuals with cancer, neutrophils may instead become prominent disease promoters, contributing to important steps during tumor progression such as angiogenesis and metastasis (4). In addition to the classical antimicrobial roles of neutrophils mentioned above, formation of neutrophil extracellular traps (NETs) was described approximately a decade ago as a novel defense mechanism during severe bacterial infections (5). NETs are formed when activated neutrophils externalize their chromatin and granular content and form a meshwork of DNA strands that function as a trap for microbes. In fact, a cell death process in neutrophils different from apoptosis and necrosis, and similar to what we today refer to as NETosis, was described already 1996 (6). The initial description of NET formation (NETosis) as a response to bacterial infections has now been followed by reports of NETs in infections caused by viruses and fungi (7–20) but also in sterile inflammation during conditions such as atherosclerosis, diabetes, and systemic lupus erythematosus (SLE) (21–24). Interestingly, NETosis was also detected in individuals with cancer for the first time a few years ago (25, 26), and the consequences are only beginning to emerge. Platelets have been found to play an essential role as inducers of intravascular NETosis in response to lipopolysaccharide (LPS) (27, 28). Conversely, NETs provide a strong activation signal for platelets due to the externalized DNA and associated histones, promoting platelet aggregation and thrombosis (29). This review describes mechanisms behind tumor-induced NETosis with a special focus on neutrophil–platelet interactions in an individual with cancer. Furthermore, consequences of tumor-induced NETosis and possible therapeutic approaches to target NETs in cancer patients will be discussed.

## Mechanisms of NETosis

During NETosis, activated neutrophils release their chromatin and granular content and form a web-like structure from strands of DNA that functions as a trap for infectious agents in the circulation (30). Secretion of neutrophil-derived proteases, such as neutrophil elastase (NE) and myeloperoxidase (MPO), contributes to a locally elevated concentration of antimicrobial substances and hence enables efficient destruction of pathogens. Both nucleic acids and the associated histones are potent inducers of platelet activation and therefore exert a prothrombotic effect with platelet aggregation and fibrin deposition as a result. So how can neutrophils form these extracellular traps? Current knowledge suggests that NETosis can occur either as a cell death-associated mechanism or in a vesicular-dependent manner where the neutrophil survives and continues to function after NET formation (referred to as “vital NETosis”). In the case where NETosis results in neutrophil death, the suggested process is dependent on chromatin decondensation, degradation of the nuclear membrane, and cellular lysis with associated release of chromatin and granular contents into the extracellular space. Nuclear decondensation is initiated by epigenetic modifications of histones, citrullination (i.e., arginine converted into citrulline), mediated by the enzyme peptidyl arginine deiminase 4 (PAD4). PAD4 has proven to be required for NETosis to be initiated and neutrophils in PAD4-deficient mice lack ability to form NETs (31, 32). Degradation of the nuclear membrane is driven by NE, which has to be translocated to the nucleus for this purpose (33). Furthermore, isolated neutrophils deficient in MPO fail to form NETs, suggesting that MPO is required for NETosis (34). In contrast to vital NETosis, which has been described as a quick event, the process of lytic NETosis takes several hours to complete (30). In addition, a recently identified process of programmed cell death, necroptosis, was earlier this year implicated as an additional mechanism for NETosis (35). Necroptosis is associated with inflammation and has been suggested to be involved in inflammatory conditions such as Crohn’s disease (36). The mechanism for necroptosis-associated NETosis was shown to depend on activation of the mixed lineage kinase domain-like protein (MLKL) for membrane degradation and subsequent cell death (35). The process of vital NETosis was first described a few years ago (30, 37). During infectious conditions, vital NETosis occurs upon stimulation of TLRs by both gram-negative and gram-positive bacteria, and involves nuclear envelope blebbing and vesicular trafficking of DNA to the extracellular space (37). The process leaves the cell membrane intact and allows the neutrophil to continuously exert its classical function *via* protease release and phagocytosis. Whether both cell death-associated and vital NETosis occur in individuals with cancer is still not clear.

It has been reported that only a fraction of all neutrophils are capable of forming NETs (30). How to distinguish these specific neutrophils with capacity for NETosis is still not clear. It has been suggested that the ability to form NETs is related to aging of the neutrophil, a process paralleled with upregulation of CXCR4 on the cell surface (38). Interestingly, the same study demonstrates that the aged neutrophil population is expanded under pathological conditions. It was recently suggested that the lifespan of a neutrophil may be significantly longer than previously reported and that the average human neutrophil remains in the circulation for more than 5 days (39). Therefore, the population of neutrophils that form NETs may be larger than previously expected. However, the finding of an extended lifespan of neutrophils beyond 1 or 2 days has been questioned (40). A vast amount of studies further support that neutrophils indeed are more heterogeneous than earlier presumed. For example, migration of neutrophils has previously been described as a one-way transfer from the circulation into the tissue. However, several publications now report observations of reversed migration of tissue-resident neutrophils back into the vasculature (41–43). Furthermore, a polarization similar to that of macrophages has been suggested for neutrophils with a division into antitumorigenic neutrophils (N1) and protumorigenic neutrophils (N2) (44). Sagiv and colleagues recently demonstrated that cancer is associated with a switch in neutrophil phenotype towards a low-density neutrophil type with more immature appearance and less lobulated nuclei (45). This subpopulation of neutrophils was suggested to be protumorigenic, as compared to high-density neutrophils with an antitumorigenic function. How this relates to NETosis was not discussed in the paper. However, low-density neutrophils have previously been isolated from patient with SLE, an autoimmune disease characterized by NETosis (46, 47). These cells were further verified to be highly prone to undergo NET formation (48), suggesting that the protumorigenic neutrophils identified by Sagiv and colleagues are indeed a potential source of NETs. Studies of NETosis in autoimmune disease have suggested a role for proteinase 3 (PR3) in NET induction (49). In small-vessel vasculitis, antineutrophil cytosplasmic antibodies (ANCA), and specifically those directed against PR3, were demonstrated to induce NETosis (50). Whether PR3 stimulation mediates NETosis also in malignant disease is not yet known.

## The Role of Platelets in NETosis

**Figure 1:**
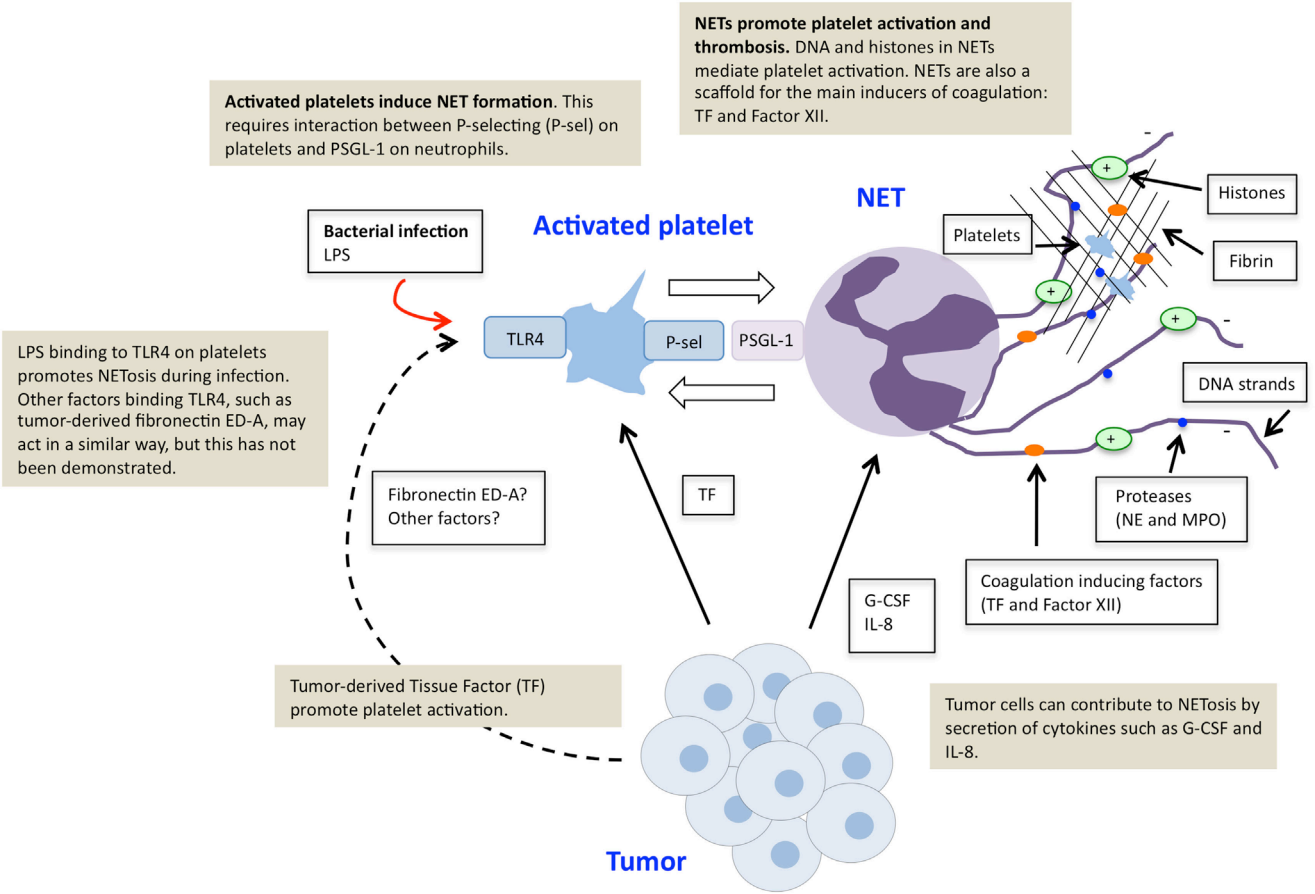
**Platelet–neutrophil crosstalk in tumor-induced NETosis**. Tumor cells can directly induce NETosis in an individual with cancer by secretion of factors such as G-CSF and IL-8. Tumors furthermore promote platelet activation, for example, by production of tissue factor (TF). Activated platelets function as inducers of NETosis. This effect is mediated *via* direct binding of P-selectin on activated platelets and PSGL-1 on neutrophils. Stimulation of platelets *via* toll-like receptor 4 (TLR4), by LPS during infectious disease or tumor-derived factors, possibly fibronectin ED-A, may further contribute to platelet-induced NETosis. NETs further stimulate platelet activation and thrombosis due to externalized chromatin and localization of TF and factor XII.

LPS, a component of the cell wall in bacteria, is an inducer of NETosis during infectious disease (27). However, bacteria-derived LPS is not a general cause of NETosis in individuals with cancer, unless the patient suffers from bacterial infection. So how can a tumor induce NETosis? While some mechanisms for tumor-induced NETosis have been described, there are possibly others that remain to be identified. A summary of identified mechanisms can be found in Figure 1. The first report of NETs in cancer appeared a few years ago and demonstrated that presence of a tumor primed neutrophils to undergo NETosis (25). The authors suggested that G-CSF was a critical factor for tumor-induced NETosis in mice with cancer. The importance of G-CSF was recently confirmed in another study, where tumors expressing high levels of G-CSF were demonstrated as more powerful inducers of NETosis than tumors expressing low levels of G-CSF (51). Furthermore, inhibition of G-CSF by injection of anti-G-CSF antibodies efficiently suppressed NET-induced vascular dysfunction in distant organs of mice with mammary carcinoma. It is however likely that additional factors are involved in induction of tumor-associated NETosis. The cytokine IL-8, frequently expressed by various tumor cells, has, for example, been described as a NET-inducing factor and was recently demonstrated to be crucial for tumor-induced NETosis (5, 52).

As mentioned, activated platelets can regulate NET induction, although NETosis can also occur independent of platelet interaction, as exemplified by PMA stimulation. An important interaction for this effect seems to be the binding of P-selectin on activated platelets to PSGL-1 on neutrophils. It was recently demonstrated that platelets from P-selectin-deficient mice failed to induce NETosis, while platelets from mice with increased P-selectin levels were more prone to induce NETs upon co-culture with neutrophils (53). Platelets have previously been described as sensors during infectious disease for the severity of an infection, where LPS binding to TLR4 on the platelet surface is essential to determine whether NETosis should be initiated (27). Although LPS-induced TLR4 activation does not take place as a result of a tumor, it is possible that other tumor-derived factors can activate platelets *via* TLR4. For example, it has been shown that a tumor-associated splice variant of fibronectin, extradomain A (ED-A), can bind to TLR4 (54). Interestingly, studies in mice recently demonstrated that signaling *via* TLR4 on platelets by fibronectin ED-A promotes platelet aggregation and arterial thrombosis (55). Whether this effect is mediated *via* NETosis was not addressed, but the possibility is briefly discussed in the paper. Furthermore, these studies were not performed in a cancer setting, and the relevance of fibronectin ED-A for cancer-associated thrombosis still remains to be determined.

The importance of platelets for NET induction is obviously not a tumor-specific phenomenon. However, it has been known for more than a century that individuals with cancer suffer from increased risk for thrombotic disease – a fatal consequence of enhanced platelet activation (56, 57). The hyperactive state of platelets in malignant disease has been attributed to the fact that many tumors express Tissue Factor (TF), which leads to thrombin formation, coagulation, and platelet activation (58). Enhanced platelet activation in cancer patients does not only contribute to thrombosis but also to malignant progression by promoting processes such as tumor angiogenesis and metastasis (59). The increased platelet activation in cancer patients could therefore be a contributing factor to enhanced NETosis during malignant disease.

## Other Types of Platelet–Neutrophil Interactions

While the specific interplay between platelets and neutrophils in formation of NETs was quite recently discovered, interactions between platelets and neutrophils were described much earlier. Already 50 years ago, the phenomenon of platelets adhering to neutrophils was described and referred to as platelet satellitism (60–64). These platelet–neutrophil complexes were observed in a number of pathological conditions, but their contribution to disease was not clear. Interestingly, a case study from 1975 described platelet–neutrophil aggregation in a patient with invasive prostate cancer, but the cause or significance of the finding was not further explored (65). Today, complex formation between platelets and neutrophils are known to occur and contribute to a wide variety of pathological conditions, such as asthma, ulcerative colitis, sepsis, rheumatoid arthritis, and acute coronary syndrome (66–75). By which mechanism do platelet–neutrophil complexes form? Initial platelet–neutrophil aggregation is mediated mainly by binding of the surface receptor P-selectin on activated platelets to neutrophil PSGL-1 and results in activation of the neutrophil (76–79). Thereafter, integrin receptors are important for continuous platelet–neutrophil interactions. For example, Gp1b-IX-V and alpha-IIb-beta-3 (GpIIb/IIIa), *via* fibrinogen, mediate binding to integrin alpha-M-beta-2 (Mac-1) on the neutrophil, while integrin alpha-L-beta-2 (LFA-1) on neutrophils can adhere to platelets *via* ICAM-2 (80–83). Platelets also facilitate leukocyte adherence to the endothelium *via* the same interactions, for example, upon damage to the vessel wall when direct adherence of leukocytes to endothelial cells is compromised and platelets function as a bridging factor (81). The ability of platelets to regulate neutrophil function is not limited to NETosis. It has been demonstrated that platelets promote initiation of inflammation by regulating neutrophil crawling, an effect dependent on signaling *via* PSGL-1 (84). Activated platelets can also promote neutrophil degranulation and phagocytosis (27, 85). Moreover, platelet-derived soluble CD40L promotes formation of reactive oxygen species (ROS) in neutrophils, which contributes further to the antimicrobial effect (86). There are also evidence showing that interaction between platelets and neutrophils promote metastasis by formation of an early metastatic niche (87). This study by Labelle et al. demonstrated that granulocyte recruitment to the metastatic site is mediated by platelet-derived CXCL5 induced by contact with tumor cells and signaling *via* the CXCR2 receptor on granulocytes. If the interaction between platelets and granulocytes is blocked with a CXCR2 antibody, metastatic seeding is significantly impaired. This study further highlights the importance of platelets as critical regulators of neutrophil function.

## Consequences of Tumor-Induced NETosis

What are the consequences of NETosis in individuals with cancer? The data presented so far suggest that tumor-induced NETosis may be a promoter of cancer-associated pathology. A couple of studies show that NETs may directly contribute to malignant progression. For example, Cools-Lartigue and colleagues showed that infection-induced NETs contribute to metastasis by sequestration of tumor cells in the circulation of mice with cancer (88). This suggests an increased risk for metastasis if cancer patients are affected by infectious disease. Recently, direct cancer-promoting effects were further demonstrated in a study where NETs were suggested to contribute to tumor relapse after surgery in patients with metastatic colorectal cancer. While this study did not address tumor-induced NETosis directly but rather NETosis induced by surgical stress, it still highlights the possibility that NETs could contribute to tumor progression and relapse (89). This finding is in line with an earlier study, suggesting that presence of NETs in tumor biopsies correlated with relapse in patients with Ewing sarcoma (26). Besides direct effects on malignant progression, tumor-induced NETosis further contributes to systemic pathological effects of cancer. For example, NETs have been suggested to promote cancer-associated deep vein thrombosis (25). Hence, the interaction between neutrophils and platelets in NETosis is not limited to platelet-induced of NET formation, but NETs can also stimulate platelet activation – adding yet an important aspect to the complex interplay between platelets and neutrophils. The procoagulant effect of NETs is primarily mediated *via* the negatively charged DNA inducing the intrinsic pathway of coagulation (90) and by histones contributing to thrombin formation (91). Moreover, both TF and factor XII, inducers of the extrinsic and intrinsic coagulation pathways, respectively, can be found in NETs (92–94). Furthermore, it was recently demonstrated that NETosis contributes to impaired vascular function and systemic inflammation in organs that are not sites for tumor growth, such as heart and kidneys, in mice with mammary carcinoma and insulinoma (51). When mice were treated with DNase I to dissolve NETs, vascular function was restored and inflammation abolished. Hypoperfusion of the renal vasculature and associated inflammation are indicators of renal insufficiency – a frequent issue in cancer patients with mortal consequences (95–97). Whether suppression of NETosis could prevent renal insufficiency in individuals with cancer remains to be explored. NETosis was connected to both thrombosis and vascular dysfunction in a study published earlier this year (98). Analysis of blood and post-mortem tissues from ischemic stroke patients revealed that a high number was affected by known or occult cancer and that this could be associated with formation of arterial microthrombi with presence of NETs in various organs. Altogether, these studies suggest that tumor-induced NETosis is connected to poor prognosis in cancer patients. It is however likely that the consequences of tumor-induced NETosis are not limited to those described today, but more reports on this phenomenon should be expected.

## Therapeutic Targeting of NETosis in Individuals with Cancer – What Are the Options?

The role of tumor-induced NETs as potential promoters of malignancy and associated complications, such as thrombosis and systemic inflammation, suggests that therapeutic approaches to suppress NETosis might be beneficial for cancer patients. Several potential strategies could be considered for this purpose. Treatment with DNase I, a strategy to degrade extracellular DNA strands, would be an option to dissolve already formed NETs. DNase I is already in clinical use for treatment of patients with cystic fibrosis, which indicates its safety as a drug (99). Another option would be to prevent NETosis by inhibition of PAD4, an enzyme required for initiation of NETosis (32). Specific PAD4 inhibitors, with capacity to prevent formation of NETs from both human and murine neutrophils, were recently developed (100). A third alternative approach would be treatment with heparin, which function to destabilize NETs by extraction of histones (29). Heparin has long been used in the clinic for its anticoagulative effects and is therefore well established as a therapeutic method. Based on current knowledge about NET induction described in this review, intervening with the P-selectin/PSGL-1 interaction could be yet a potential therapeutic strategy. An important issue to address to enable clinical use of NET targeting approaches is whether there are risks with NET inhibition. A few studies performed in mice lacking PAD4 and hence unable to form NETs have been published but with various results. While increased susceptibility to bacterial infection was described as a consequence of PAD4 deficiency in one study, other studies reported that mice lacking PAD4 are not more sensitive to infections. Instead, it was suggested that PAD4-deficient mice are protected against septic chock (31, 32, 101). Further research is needed to fully explore the potential risks with therapeutic approaches targeting NETs.

The existing data on tumor-induced NETosis strongly indicate that targeting NETs could be beneficial for cancer patients. NETs, originally identified as a defense against severe infectious disease, seem rather to have a negative influence during malignant disease by promoting mortal processes such as thrombosis, systemic inflammation, and cancer relapse. With this in mind, NETs could provide excellent targets for future anticancer therapies, with capacity to suppress processes contributing to the absolute majority of cancer-related deaths.

## Author Contributions

A-KO and JC performed the literature search and wrote the manuscript.

## Conflict of Interest Statement

The authors declare that the research was conducted in the absence of any commercial or financial relationships that could be construed as a potential conflict of interest.
